# Advancing resilience science through cross-domain integration: Proceedings of an NIH workshop

**DOI:** 10.1016/j.neubiorev.2026.106660

**Published:** 2026-03-24

**Authors:** Stefan M. Pasiakos, Marcas M. Bamman, Stephanie H. Cook, Jonathan M. DePierro, Jacob M. Haus, Harris R. Lieberman, Karen A. Litwa, Mary M. McDermott, Herman A. Taylor, Michael Ungar, Barbara E. Cohen

**Affiliations:** a Pennington Biomedical Research Center, Baton Rouge, LA 70808, USA; b National Institutes of Health Office of Dietary Supplements, Bethesda, MD 20817, USA; c Florida Institute for Human and Machine Cognition, Pensacola, FL 32502, USA; d School of Global Public Health, New York University, New York, NY 10003, USA; e Icahn School of Medicine at Mount Sinai, New York, NY 10029, USA; f School of Kinesiology, University of Michigan, Ann Arbor, MI 48109, USA; g Military Nutrition Division, US Army Research Institute of Environmental Medicine, Natick, MA 01760, USA; h Brody School of Medicine, East Carolina University, Greenville, NC 27834, USA; i Feinberg School of Medicine, Northwestern Medicine, Northwestern University, Chicago, IL 60611, USA; j Cardiovascular Research Institute, Morehouse School of Medicine, Atlanta, GA 30310, USA; k Resilience Research Centre, Dalhousie University, Halifax, NS, Canada; l Collaborative Consultants, Inc., contracted through ICF International in support of the National Institutes of Health Office of Dietary Supplements, Bethesda, MD 20817, USA

**Keywords:** Cross-domain metrics, Mechanistic stress response, Multilevel systems resilience, Psychosocial resilience, Physiological resilience

## Abstract

The National Institutes of Health (NIH) Office of Dietary Supplements hosted a workshop titled *Advancing the Biomedical Science of Resilience: A Discussion of Measures and Metrics* on September 24–25, 2024. The workshop convened NIH program staff and researchers from academia and federal agencies to propose and identify measures and metrics that capture protective factors contributing to resilience across the lifespan. Various perspectives on resilience were thoroughly discussed, including those related to cellular, physiological, psychosocial, community, and environmental domains, reflecting the complexity and multidimensionality of resilience science. Central to the workshop were discussions to examine characteristics of resilience studies across domains and to explore outcome-specific measures and metrics and study designs needed to validate those outcomes and accurately measure resilience in biomedical research. The workshop concluded with a panel discussion on the challenges of advancing the science of resilience and the need for robust frameworks for studying the concept. This article presents the proceedings from that workshop and outlines strategies for advancing and strengthening the science of resilience through greater integration across research domains. Leadership in convening diverse perspectives and fostering multidisciplinary dialogue, such as through consensus-building activities and collaborative forums, will be important for advancing shared frameworks and understanding resilience across the lifespan.

## 1. Introduction

Resilience, an active field of study since the mid-twentieth century, has evolved considerably in its conceptualization. Initially regarded as a trait, resilience is now understood as a dynamic process that extends beyond the individual to encompass community and environmental factors (Rugh and Wolff, 1955; Garmezy, 1974; Werner and Smith, 1982; Rutter, 1985; Fleming and Ledogar, 2008). Definitions of resilience have also progressed, yet often they remain narrowly focused and specific to individual systems or domains (Cicchetti and Garmezy, 1993; Hadley et al., 2017; Taylor et al., 2022; Wolke et al., 2025). To unite these concepts, the National Institutes of Health (NIH) has defined resilience as the capacity of a system to resist, adapt, recover, or grow from a challenge (Brown et al., 2023a).

**Fig. 1.**
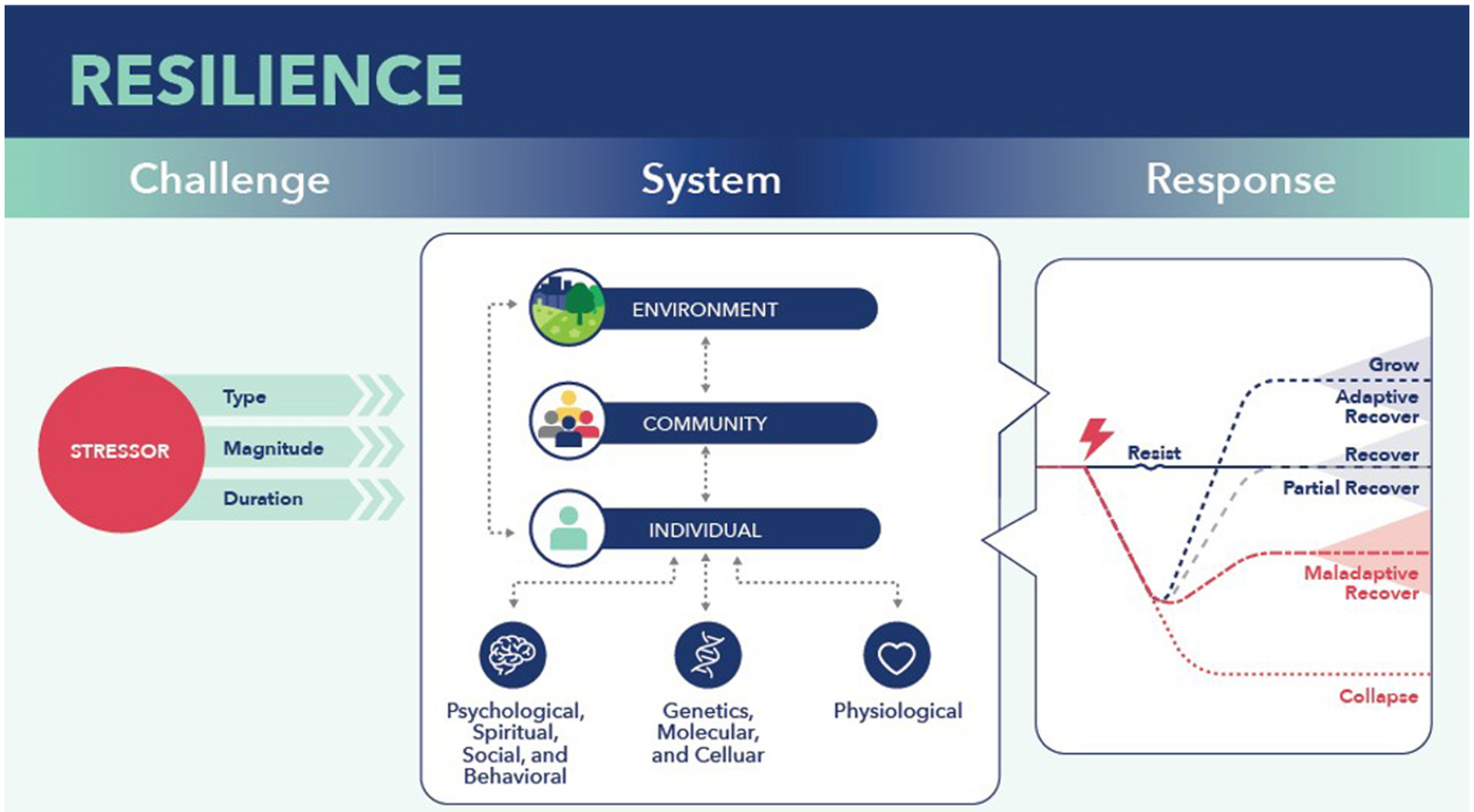
NIH Resilience Concept Model. The proceedings presented in this paper from the NIH workshop titled *Advancing the Biomedical Science of Resilience: A Discussion of Measures and Metrics* reflect the concept of resilience as presented in the NIH Resilience Concept Model (figure adapted from Brown et al., 2023a). It illustrates that over time, a system’s response to a challenge might show varied degrees of reactions that likely fluctuate in response to the type of challenge, severity of the challenge, the length of time exposed to the challenge, and/or innate or intrinsic factors. The system can be represented by various domains, factors, or processes that impact human health, including (but not limited to) environmental or community exposures as well as an individual’s cellular, molecular, or genetic (i. e., mechanistic), physiological, and psychological, spiritual, social, or behavioral (i.e., psychosocial) capacity. Reactions of resistance, recovery, adaptive recovery and growth, all equivalent to or above baseline, are considered resilience outcomes.

According to NIH, the science of resilience can be categorized into three broad domains: individual, community, and environment, all of which influence each other. The individual domain can be subdivided into systems: 1) cellular, molecular, or genetic, 2) physiological, and 3) psychological, spiritual, social, or behavioral. For purposes of this article, mechanistic will collectively refer to cellular, molecular, or genetic systems, and psychosocial to psychological, spiritual, social, or behavioral systems. The NIH resilience conceptual model (Fig. 1) shows that a system’s response to a challenge may vary depending on the severity of the challenge, duration of exposure, and, in the case of the individual, innate biological factors (Brown et al., 2023a; Reed et al., 2024). Specifically, if exposure to a challenge results in no change, the system has demonstrated resistance. If the system initially declines but returns to baseline, it has recovered. If the system not only recovers but also improves from baseline, this indicates growth or adaptation. Each of these responses constitutes resilience. Conversely, when a challenge leads to persistent perturbation, degradation, or failure of a system to recover, resilience is absent, indicating an adverse response or collapse.

An NIH resilience research design tool provides a harmonized research model that can be used to guide the study of resilience across all domains (Brown et al., 2023b). It focuses on identifying and understanding factors that strengthen or protect despite stress exposure and uses metrics designed to capture changes that advance the system to resistance, recovery, growth, or adaptive responses. However, while the NIH definition of resilience, its conceptual framework, and research design tool represent significant advances, the science of resilience continues to have challenges. A key area of confusion is the lack of consensus on what differentiates resilience science from traditional biomedical fields, such as disease prevention. It also remains unclear whether resilience can be mechanistically attributed to novel factors or protective pathways, or to those already well characterized. Critically, how the mechanisms of resilience can be accurately and reliably studied within and across domains remains uncertain. Finally, while there remains ambiguity about the relationship between a stressor and a resilience outcome (Brown et al., 2023a), exposure of a system to a stressor or challenge is a prerequisite to measurin*g* the resilience of a system. It is important to note that discussions on the measurement of resilience focus on a stressor or a challenge that is considered a stimulus that threatens homeostasis, as opposed to a stress response, which is the reaction of the system to a stressor as defined by Koolhaas et al. (2011). Characterization of stressors in resilience research is critical and includes their magnitude, whether they are chronic or acute, and the life cycle or life stage at which the stressor might impact any given system.

In 2024, both the NIH National Heart, Lung, and Blood Institute (NHLBI) and the National Institute on Aging (NIA) held workshops on resilience within their respective fields. The NHLBI workshops focused on the impact of sleep on cardiovascular resilience and on the resilience of lung progenitor cells, including applications in early diagnosis, prevention, and treatment of chronic lung diseases (NHLBI, 2024a; NHLBI, 2024b). The NIA workshop centered on three factors: the biology underlying resilient outcomes; the social, environmental, genetic, and psychosocial factors that influence that biology; and the biomarker testing and imaging that predict resilient outcomes for older adults (Colón-Emeric et al., 2024).

Building upon these workshops, the NIH Office of Dietary Supplements and the NIH Resilience Research Working Group convened a workshop in November 2024 titled *Advancing the Biomedical Science of Resilience: A Discussion of Measures and Metrics*. The workshop brought together NIH program staff with broad representation across Institutes, Centers, and Offices, as well as leading resilience researchers from academic institutions and other federal agencies, to explore the cellular, physiological, psychosocial, community, and environmental factors that contribute to resilience. The workshop was designed to define measures and metrics within and across resilience domains, and to identify the study designs needed to validate those measures and metrics that accurately measure resilience. Barriers towards and opportunities for advancing the field were also examined.

The workshop began to answer some of the key unanswered questions raised at the NHLBI and NIA workshops on resilience biomarkers with a focus on temporal issues, measurement assays, and specific biomarkers. Most importantly, the workshop began to address the cross-domain aspects of resilience – not only identifying metrics that can be used across domains but also understanding the practical implications of a more holistic approach to resilience measurement. Using inflammation markers and metrics as an example of this approach, multiplex cytokine assays may identify inflammation (a loss of resilience) at the cellular and physiological levels, predicting elevated psychological stress or loss of resilience. It may also identify the need to assess community and environmental factors that may be influencing inflammation at the individual level, such as water pollution (environmental) or antiquated water or sewage systems (community). This supports the cyclical nature of resilience as presented in Fig. 1, with resilience or lack of resilience in one domain or subdomain impacting resilience outcomes in other domains.

Herein, we present the proceedings from the workshop, organized by domain: individual (i.e., mechanistic, physiological, and psychosocial), community, and environment, with a focus on domain-specific measures and metrics along with opportunities and challenges for advancing a validated set of resilience measures and metrics within the domain. The article concludes with a section exploring resilience research gaps and how domain-specific measures and metrics might be used across domains to provide a more robust framework for studying resilience.

## 2. Individual resilience domain

### 2.1. Measures and metrics of mechanistic resilience

As the smallest of living systems but one that impacts all higher levels of organization, we first consider mechanistic resilience that incorporates individual genes, molecules and cells. With limited exception, this discussion is primarily focused on the biology of human cells and is largely agnostic to tissue origin or cell type, as established measures and metrics of cellular function and resilience apply to and can be tested in most any mammalian cell. Within this framework, two temporal scales are considered: (i) resilience to an acute/short-term stressor; and (ii) chronic resilience, which, for this article, is anchored to cellular biological hallmarks of aging. Discussion of the aging/chronic temporal scale extends logically to tissue-level and systemic measures and metrics of resilience – the organizational levels required to assess some key biological hallmarks of aging. In all cases, a central premise of this resilience discussion, in line with the NIH definition, is that there are essentially two phases of resilience that can be assessed: (i) responses during a stressor/challenge (resistance, adaptation); and (ii) responses following a stressor/challenge (recovery, growth). Mechanistic stress resilience is fundamental to our understanding of resilience at all higher levels of organismal organization (tissue – organ – multi-organ system – whole organism) and thus provides a foundation for physiological resilience—discussed in the next section of this article.

For acute studies of mechanistic (genetic, molecular, cellular) stress resilience, some robust and often used stressors include hypoxia, nutrient deprivation, hyperthermia, inflammation, and chemical/toxin exposure. Each serves as a short-term stressor that enables researchers to quantitatively measure resilience metrics both during and after stress. Measures and metrics of mechanistic resilience are often assessed in response to a stressor applied in vitro but can also be assessed on cells ex vivo to quantify the impact of an acute stressor experienced by the whole organism (e.g., measures of cellular stress/resilience in primary peripheral blood mononuclear cells following whole-body exposure to a high-altitude/hypoxic environment). Whether in vitro or ex vivo, robust measures of cellular stress resilience across common stressors are: (i) the endoplasmic reticulum (ER) unfolded protein response (UPR) stress assay; (ii) integrated stress response assay; and (iii) measures of apoptosis (Oslowski and Urano, 2011; Pakos-Zebrucka et al., 2016).

Within the hypoxia or oxidative stress domain, there are numerous approaches to measuring oxidative stress; thus, the one(s) selected are driven by the specifics of a research question. The complexity of oxidative stress necessitates an analysis that extends beyond a single pathway. In line with this, authors of a recent review recommend a multi-pronged approach for a more comprehensive assessment that would include measures of reactive oxygen species dynamics, antioxidant defense systems, and mechanistic indices of oxidative damage (Poljšak et al., 2025). If the acute stressor of interest is nutrient deprivation, informative metrics include assessments of protein turnover/proteostasis, cellular proliferation/growth, and nutrient sensing/signaling. Assessing proteostasis in the context of resilience to nutrient deprivation requires measuring multiple components of both the protein synthesis and protein breakdown machineries, as summarized by Sala et al. (2017).

Effects of acute exposure to high temperature are often assessed by evaluating heat shock protein expression and activity along with the integrated stress response assay. The impact of acute inflammation on cells is most directly measured by cytokine signaling assays that extend from receptor-ligand binding to ultimately transcription factor activation and downstream effects on gene expression and cellular function. All the aforementioned measures are also relevant when studying acute responses to chemical/toxin exposures, as are assays of DNA damage. ToxProfiler is a new approach to studying in vitro or ex vivo cellular stress resilience in the face of chemical/toxin exposure (Ter Braak et al., 2024). This in vitro reporter assay enables researchers to decipher mechanisms of action at single-cell resolution by measuring the activity of seven major cellular stress response pathways (oxidative stress, cell cycle stress, endoplasmic reticulum stress, ion stress, protein stress, autophagy, and inflammation).

For the discussion of chronic resilience, we focus on 13 biological hallmarks of aging. This aging/chronic temporal scale extends logically to tissue-level and systemic measures and metrics of resilience to assess some key biological hallmarks of aging. In each of the 13 aging hallmarks discussed, resistance to the typical aging-related decline or dysfunction in any one of these hallmarks provides an indication of resilience. Nine biological aging hallmarks can be assessed at the mechanistic level as highly informative indices of cellular aging, again with any level of resistance indicative of resilience: genomic instability, telomere attrition, epigenetic alterations, loss of proteostasis, disabled macroautophagy, deregulated nutrient sensing, mitochondrial dysfunction, cellular senescence, and stem cell exhaustion. Several viable and proven methods are available to assay each of these in vitro or ex vivo, as summarized in Tables 1 and 2. Genomic instability as a core of biological aging and disease is linked to epigenetic changes (histone modifications, hyper/hypo DNA methylation patterns), chromatin remodeling, DNA damage accumulation and blunted repair capacity, and telomere shortening, among other mechanisms (López-Gil et al., 2023). Telomere length and epigenetic clocks (Suglia et al., 2025) estimate biological age and may reflect cumulative exposure to stress. Shorter telomeres have been associated with earlier onset of chronic disease and lower survival rates in older adults (Cawthon et al., 2003; Ludlow and Roth, 2011), while newer methylation-based age predictors correlate strongly with all-cause mortality and have been validated in diverse cohorts (Levine et al., 2018). Quantitative metrics such as the Genomic Instability Metric and Genomic Instability Score were developed and are currently leveraged in the context of cancer tumor assessment and progression; yet these or similar quantitative indices may also prove useful in studies of biological aging and resilience.

Extending to the tissue level, key hallmarks of aging to study in the context of resilience include extracellular matrix changes and altered intercellular communication. Changes to extracellular matrix are commonly assessed via immunofluorescence microscopy (collagen, elastin, hyaluronan) and assays to assess mechanisms of remodeling (e. g., enzyme assays for matrix metalloproteinase), while changes in intercellular communication leverage approaches such as gap junction communication assays, ligand-receptor interactions, exosome transfer assays, or the novel CellChat platform (Jin et al., 2025). Studies of intercellular communication via extracellular vesicles (EVs) such as exosomes have boomed in recent years, including the emergence of novel mitochondrial EVs (mitoEVs), which may play a key role in biological aging and/or resilience to it (Iorio et al., 2024). Sequencing the nucleic acid cargo (e.g., long RNAs, miRNAs) or protein cargo in EVs can be quite useful in studies of intercellular communication, particularly if additional approaches are leveraged to identify the cell/tissue source(s) of the EVs (e.g., surface protein profiling, exLR-seq) (Kibria et al., 2016; Li et al., 2020). At the systemic level, two aging hallmarks that in many ways can be considered systemic indices of cellular aging are chronic inflammation and dysbiosis. Chronic inflammation is often assessed via multiplexed cytokine arrays, while dysbiosis assessments typically involve a PCR-based microbiome assessment with some tests yielding a dysbiosis index score (e.g., GA-map Dysbiosis Test) that can be useful in tracking the state of dysfunction and/or efficacy of an intervention.

### 2.2. Challenges and opportunities for advancing validated mechanistic resilience metrics

As in any study of resilience, the measures and metrics selected to assess mechanistic resilience are context dependent. Biological studies of human resilience to this point have focused largely on physiological measures and metrics, whether for studies of acute stress or more chronic exposures or conditions. We therefore propose a grand challenge and opportunity for the human resilience field that will leverage novel and potentially high-impact research strategies at the cellular level. First, the various acute stress models discussed herein can be applied ex vivo / in vitro to primary human cells isolated from blood or tissue biopsies that are collected from, for example, humans deemed resilient or less resilient in vivo in response to a given stress or challenge. This would enable mechanistic studies in a directly relevant biological system. Second, instead of testing primary cells, blood serum or isolated extracellular vesicles from humans deemed resilient or non-resilient in a given human experiment can be used to treat standardized cells in a follow-up in vitro acute stress experiment as a means of identifying potential mechanisms of resilience external to the cell proper (e.g., intercellular communication, inflammation, etc.). Third, to pursue mechanisms of chronic cellular resilience, many of the pathways and hallmarks discussed could be tested in parallel experiments of primary cells isolated from humans of the same chronological age but varying in biological age (e.g., via intrinsic capacity scores). And fourth, these same primary cultures could be tested in the aforementioned models of acute stress resilience and directly compared to cultures from younger adults. Overall, there are countless, fruitful opportunities to advance the understanding of mechanistic resilience in humans, even with the limitations imposed by studying humans as the model system.

Measures of mechanistic resilience provide a foundation for understanding how systems respond to challenge. Indicators of stress resistance, recovery, and adaptation—such as proteostasis, mitochondrial function, and inflammation—have clear analogs in physiological responses observed at the organ and whole-body level. Examining how mechanistic responses scale to physiological outcomes offers an important opportunity to connect mechanisms with functional resilience and to identify cross-domain metrics that reflect shared adaptive processes.

### 2.3. Measures and metrics of physiological resilience

Physiological resilience encompasses cardiorespiratory, metabolic, neuromuscular, endocrine, and sleep subdomains, with metrics that can be delineated into acute, chronic, and long-term scales (Table 3). The physiological domains highlighted herein reflect the principal systems discussed during the workshop and are presented as illustrative examples of measurable resilience trajectories rather than an exhaustive catalog of all potential physiological metrics. Critically, physiological resilience cannot be interpreted independently of the stressor. The magnitude, duration, and qualitative nature of a challenge define the guard rails within which resistance, recovery, or adaptation occur. A standardized exercise test, acute sleep deprivation, or a social-evaluative stressor provides controlled perturbations that enable clear quantification of response trajectories. In contrast, real-world stress exposures are heterogeneous, requiring parallel measurement of both physiological response and stressor characteristics to meaningfully quantify resilience.

Determinants of physiological resilience include baseline functional reserve, prior stress exposure, behavioral factors (e.g., sleep, nutrition, physical activity), age-related biological changes, and underlying cellular integrity. Importantly, resilience metrics must distinguish between absolute performance capacity and adaptive efficiency. For example, high cardiorespiratory fitness reflects reserve, whereas rapid recovery kinetics reflect adaptive regulation. Both contribute to resilience but represent distinct physiological constructs. Although multiple physiological systems contribute to resilience, we use cardiovascular metrics as an illustrative example given their extensive validation, clarity of interpretation, and strong linkage to acute and long-term outcomes. Acute physiological resilience metrics assess the immediate stress response. Validated and noninvasive cardiovascular metrics include heart rate and heart rate recovery (HRR), which evaluate cardiovascular re-stabilization after exertion. HRR refers to the rate at which heart rate declines during the first 1–2 min post-exertion, reflecting parasympathetic (vagal) reactivation and sympathetic withdrawal. Faster HRR is associated with greater cardiorespiratory fitness, efficient autonomic regulation, and improved prognosis across a range of health conditions. HRR after exercise has been shown to independently predict mortality risk, particularly in middle-aged adults (Cole et al., 1999).

Heart rate variability (HRV) is also used to measure the immediate stress response and refers to beat-to-beat heart rate fluctuations in time intervals between successive heartbeats and may reflect the dynamic interplay between sympathetic and parasympathetic branches of the autonomic nervous system. HRV is a controversial method; however, due to a lack of standardization across laboratories and disagreement regarding its interpretation. High HRV may indicate greater autonomic flexibility and the ability to maintain or regain homeostasis following perturbation. It has been reported to be predictive of cardiovascular outcomes in both healthy and clinical populations (Shaffer and Ginsberg, 2017). Despite limitations in standardization across laboratories, reduced HRV is an indicator of autonomic imbalance and physiological strain and is commonly interpreted as evidence of acute stress or insufficient recovery. Further, acute endocrine markers such as cortisol quantify hypothalamic-pituitary axis responsiveness, elevated post-stressor cortisol levels, and slower recovery trajectories have been linked with increased risk of cardiometabolic disease (Chida and Steptoe, 2009).

Although cardiovascular metrics provide a clear and validated model for illustrating resilience, physiological resilience is inherently multisystemic. Metabolic resilience, for example, reflects the capacity to maintain glucose homeostasis, lipid balance, and substrate flexibility in response to nutritional or energetic stress. Dynamic tests such as oral glucose tolerance testing, insulin sensitivity indices, and measures of metabolic flexibility (e.g., shifts in respiratory exchange ratio during substrate transition) quantify adaptive metabolic capacity rather than static metabolic status. Endocrine resilience similarly reflects the regulated activation and resolution of hormonal stress responses. Hypothalamic–pituitary–adrenal (HPA) axis responsiveness, assessed via cortisol reactivity and recovery trajectories, provides insight into stress sensitivity and recovery efficiency. Dysregulated or prolonged cortisol responses may signal impaired adaptive capacity even in the absence of overt disease. Collectively, these metrics assess the speed and efficiency of physiological systems in re-establishing physiological equilibrium after an acute challenge or stress.

Beyond acute perturbations, chronic physiological challenges include sustained metabolic overload (e.g., overnutrition or insulin resistance), persistent low-grade inflammation, repeated sleep restriction, caregiving burden, occupational strain, or environmental heat exposure. In these contexts, resilience may be reflected not in rapid recovery, but in maintenance of functional capacity, attenuation of cumulative physiological load, or slowed biological aging trajectories. Chronic physiological resilience metrics evaluate stability or adaptation over time, typically spanning weeks to months. ${\text{V}}^{\cdot }{\text{O}}_{2}\text{max}$, the gold standard for cardiorespiratory fitness, is a metric of metabolic reserve and a robust predictor of health. Longitudinal data from the Framingham Heart Study (Miller et al., 2024) and the Coronary Artery Risk Development in Young Adults Study (Shah et al., 2016) have established that a higher ${\text{V}}^{\cdot }{\text{O}}_{2}\text{max}$ is associated with lower cardiovascular and all-cause mortality. As a metric that integrates the functional capacity of multiple physiological systems under graded exertion and that is modifiable through behavioral interventions, and is responsive to environmental and physiological challenges, ${\text{V}}^{\cdot }{\text{O}}_{2}\text{max}$ is a dynamic metric of adaptive capacity. Blood pressure variability and maximal inspiratory pressure provide additional measurements of cardiovascular and respiratory adaptability (Joyner & Limberg, 2014; Syabbalo, 1998). Inspiratory muscle strength, a metric associated with functional reserve, has been increasingly used in prehabilitation and pulmonary resilience assessments (Langer et al., 2018).

Long-term physiological resilience metrics track physiological aging or accumulated system wear. Grip strength, supported by longitudinal evidence from the InCHIANTI cohort (Hicks et al., 2012; Cesari et al., 2015) and other aging studies (Rantanen et al., 1999; Bohannon, 2019), is a validated indicator of physiological resilience. It represents neuromuscular functional reserve and reliably predicts disability, frailty progression, hospitalization, and mortality, thereby capturing an individual’s vulnerability to future stressors and declines in adaptive capacity. Similarly, performance on the 6-minute walk test is a robust indicator of physiological resilience, with lower walk distance or accelerated decline predicting mobility disability, hospitalization, and mortality in older adults (McDermott et al., 2008). The 6MWT, therefore captures integrated cardiorespiratory and musculoskeletal reserve and reflects an individual’s capacity to withstand and recover from functional stress over time. Together, these functional capacity measures capture multisystem reserve and the cumulative impact of aging and chronic stress, providing practical and validated metrics of long-term physiological resilience.

### 2.4. Opportunities and challenges for advancing validated physiological resilience metrics

Validating physiological resilience measures and metrics requires study designs tailored to the underlying construct—resist, recover, grow, and adapt. Experimental challenge protocols, such as the Trier Social Stress Test (TSST) (Allen et al., 2016), cold pressor tests, graded exercise tests, or sleep deprivation/restriction, are frequently used to quantify acute responses. For example, HRV rebound and cortisol normalization are assessed in controlled settings to test the short-term recovery of a given system. Chronic and long-term resilience measures, such as ${\text{V}}^{\cdot }{\text{O}}_{2}\text{max}$ or frailty progression, require longitudinal designs. Mechanistic and translational studies increasingly link physiological metrics with cellular or molecular processes, such as mitochondrial biogenesis, telomere length, or epigenetic modifications, bridging multi-system resilience.

New technologies enable continuous resilience monitoring. Wearable biosensors provide real-time HRV, actigraphy, and sleep data, while ecological momentary assessment (EMA) captures context-specific stress and recovery patterns in daily life that reflect an individual’s ability to maintain or regain physiological stability in response to everyday challenges. Hybrid designs that combine technologies strengthen the ecological validity of a study while also retaining temporal resolution. In real-world studies, wearable biosensors and EMA tools enable simultaneous quantification of physiological dynamics and contextual stressor exposure. Rather than assuming uniform exposure, resilience analyses may incorporate time-varying stressor metrics (e.g., physical workload, ambient heat index, psychosocial stress ratings) and apply multilevel or within-person modeling to estimate exposure-adjusted response trajectories. This approach allows differentiation between variability due to stressor intensity and variability due to adaptive capacity.

Studies designed to test physiological metrics of resilience should use a standardized stressor to perturb the system and quantify. Resilience-oriented physiological study designs differ from conventional experimental designs in that they must incorporate a defined perturbation and repeated measurement capable of capturing response trajectories. A single post-exposure measurement cannot establish resilience. Instead, resilience requires quantification of:

1. magnitude of deviation from baseline during challenge (resistance),
2. rate and completeness of return toward baseline (recovery), and
3. long-term functional change following repeated exposure (growth or adaptation).

Importantly, resilience metrics should distinguish between physiological reserve (capacity) and regulatory efficiency (dynamic control). For example, ${\text{V}}^{\cdot }{\text{O}}_{2}\text{max}$ reflects functional reserve, whereas heart rate recovery reflects autonomic re-stabilization efficiency. Both contribute to resilience but capture different dimensions of adaptive capacity. Rigorous studies should ensure experimental control and repeatability through design elements such as standardized time-of-day for assessments (e.g., conducting all testing at the same circadian phase to minimize diurnal variation in hormones, autonomic function, and performance), as well as consistent sleep duration, dietary habits, and exercise habits. Experimental control, in this context, refers to holding these factors constant or measuring and accounting for them so that observed changes can be attributed to the stressor rather than uncontrolled biological or behavioral variability. Further, incorporation of real-time monitoring as previously described can strengthen the ecological validity of the study and capture resilience under normal everyday life conditions. Analysis tools and strategies will undoubtedly vary by the research questions themselves, but resilience-aligned study designs and metrics should always attempt to capture the magnitude of change from baseline during the stressor (resistance), the rate or time constant of return toward baseline (recovery), and the change from baseline following a single or repeated exposure (growth/adaptation). If possible, developing and validating Minimal Clinically Important Difference (MCID) scores for these measures and metrics will ensure results align with the NIH resilience framework and the best suggested study design practices.

Several challenges slow the widespread adoption of resilience measures and metrics. Inter-individual variability complicates data interpretation. ${\text{V}}^{\cdot }{\text{O}}_{2}\text{max}$ thresholds may differ dramatically by demographic group, and population-specific norms exist to facilitate between-person comparisons (Kaminsky et al., 2019). In contrast, HRV is most informative as a within-person index, where deviations from an individual’s baseline can signal elevated stress, poor recovery, or autonomic dysregulation (Shaffer and Ginsberg, 2017; Koenig and Thayer, 2016). The lack of standardization in measurement protocols further limits comparability across studies (Howley et al., 1995). For example, HRV varies by body position and device type, while ${\text{V}}^{\cdot }{\text{O}}_{2}\text{max}$ requires specialized metabolic carts, controlled laboratory conditions, and verification of maximal effort. Harmonized procedures will allow for comparability across studies and cohorts. Instability over time also poses conceptual challenges. Physiological resilience metrics are dynamic, yet most studies rely on cross-sectional designs or single-time-point measurements. Capturing recovery trajectories or adaptive gains requires repeated assessments or time-series analyses. Concept specificity is also a concern since a metric should directly reflect resilience, not just general health, disease risk, or stress exposure. The metric should capture the capacity to resist, recover from, grow, or adapt to a challenge, rather than simply showing a physiological state at a fixed point in time. Composite indices like allostatic load may reflect cumulative stress burden and, therefore, resilience, but such data should be carefully interpreted when attempting to imply that allostatic load is a metric of resilience, particularly in responses to an intervention (Guidi et al., 2021). Finally, implementation barriers exist. For example, employing assays to assess epigenetic clocks or telomere length is expensive, likely cost-prohibitive, and currently limited to specialized labs, slowing their widespread use and subsequent contributions towards advancing resilience science.

Along with these challenges, there are also many opportunities. The physiological research domain offers an array of metrics spanning acute, chronic, and long-term dimensions of resilience. Strategic use of experimental, longitudinal, and mechanistic designs is essential to validate these metrics and measures. Opportunities to overcome barriers related to standardization, variability, and practical implementation are critical for quantifying physiological resilience across domains and time scales, enabling targeting interventions that enhance adaptation, recovery, and long-term health. Physiological resilience reflects the integrated function of multiple biological systems and provides a bridge between cellular mechanisms and psychosocial outcomes. Metrics such as HRR, autonomic flexibility, and functional capacity capture dynamic responses to stress that are also closely linked to psychosocial experiences of stress, coping, and recovery. Aligning cellular, physiological, and psychosocial measures may help clarify how these processes jointly contribute to resilience trajectories over time.

### 2.5. Measures and metrics of psychosocial resilience

As described earlier, resilience is a complex construct with many definitions, most intended to address specific research domains, and across all fields is studied by first defining the response of the system or outcome of interest and then examining how that outcome responds to stressor exposure over time. The American Psychological Association (APA) defines resilience as the process and outcome of successfully adapting to difficult or challenging life experiences, especially through mental, emotional, and behavioral flexibility and adjustment to external and internal demands (APA Dictionary of Psychology, 2018). A recent comprehensive review of longitudinal resilience studies defines resilience as the process of successfully adapting to adversity, which involves mental, emotional, and behavioral flexibility in the face of changing demands (Wolke et al., 2025). The similarity of these two definitions indicates substantial consensus among scientists in the field on the definition of psychosocial resilience.

As noted above, resilience can only be assessed by examining the response of the psychosocial system over time after exposure to stressors to determine if an individual’s behavioral health is preserved, recovers, or improves relative to baseline in challenging circumstances. Since resilience is the response to stressors over time, proper assessment of psychosocial resilience requires longitudinal designs that quantify stressor exposure and outcome dynamics (Kalisch et al., 2021). In studies of psychosocial resilience, it is essential to assess the extent of stressor exposure and control for it as an essential element. While early psychological research framed resilience as a fixed individual trait, emphasizing dispositional characteristics, it is now widely recognized that resilience is a dynamic process that can include personal growth following trauma (Tedeschi and Calhoun, 2004). There are several approaches to the measurement of resilience. For example, Bonanno et al. (2011) state that most individuals display resilient trajectories and experience short-lived distress before returning to baseline, and Southwick et al. (2014) recognize that resilience integrates socio-cultural and lifespan perspectives. As such, resilience is framed as a multilevel construct shaped by environmental feedback loops, family and community resources, and systemic supports (Ehrlich et al., 2024; Masten, 2016; Motti-Stefanidi et al., 2022; Ungar, 2021). The above conceptualization of resilience, the trajectory method of Bonanno et al. (2011), can be classified as an outcome measurement method. However, there are other approaches, such as the residualization method, which focuses on deviation from expected functioning (Wolke et al., 2025).

Numerous instruments (e.g., questionnaires) have been developed to assess factors associated with resilience and typically focus on dispositional and personality-based constructs. However, unless administered repeatedly, these instruments to not measure resilience. The most widely cited instrument is the Connor-Davidson Resilience Scale (CD-RISC; Connor and Davidson, 2003), which consists of 25 items that assess perceived adaptability, persistence, and coping ability. It is often used as a baseline predictor measure and to assess changes in resilience in longitudinal intervention studies. Other tools used to assess disposition include the Grit scale (Duckworth and Quinn, 2009) and the Brief Resilience Scale (Smith et al., 2008). These three tools classify resilience as self-perceived adaptability and are useful for cross-sectional comparisons or screening populations at baseline.

Another measurement tool focuses on trainable behaviors and coping processes. The Mount Sinai Resilience Scale (DePierro et al., 2024) examines resilience factors that are trainable behaviors or means of coping that promote stress adaptation in different populations. The various metrics discussed above are suitable for measuring the impact of controlled exposure to stressors in environments such as military training, which are designed to prepare individuals for potential real-world stress exposure gradually over the course of exposure to various stressors (Morgan et al., 2001; Farina et al., 2019).

Resilience metrics can also be grouped by social and ecological systems (Theron et al., 2025). The Child and Youth Resilience Measure (CYRM; Ungar and Liebenberg, 2011; Jefferies et al., 2018) is among the most popular tools for assessing resilience in children and adolescents if repeatedly administered, as it accounts for cultural and other social ecological factors. Development of the CYRM was based on research across diverse socioeconomic groups, which assessed social-ecological aspects of protective factors and processes. It has been used in over 150 studies throughout the world (see, for example, Panter-Brick et al., 2018). Similarly, Motti-Stefanidi et al. (2022) used composite metrics to examine resilience across personal, familial, and educational systems during economic crisis, showing how systemic interactions can drive adaptive outcomes.

A related category of metrics includes developmental and relational measures. Early attachment relationships guide how individuals will cope with stress and adversity over their lifetime (Bowlby, 1979; Cook and Calebs, 2016). Disentangling these processes is difficult and thus documenting how measurements of attachment relate is important. The Experiences in Close Relationship-Revised questionnaire (ECR-RC–12; Brenning et al., 2014) measures attachment anxiety and avoidance. Godor et al. (2023) found that attachment anxiety (ECR-RC–12) was highly correlated with the sense of mastery, sense of relatedness, and emotional reactivity subscales of the Resiliency Scales for Children & Adolescents (RSCA; Prince-Embury, 2013), suggesting overlapping constructs. However, the attachment avoidance subscale was not highly correlated, indicating these may be distinctive measurements of attachment avoidance and resilience.

Many psychological constructs overlap with or contribute to resilience and are used in resilience studies. Examples include: the Profile of Mood States (Heuchert and McNair, 2012; Lieberman et al., 2016; Han and Wang, 2022), the Life Orientation Test-Revised (Scheier et al., 1994), versions of the Medical Outcomes Study Social Support Survey (Sherbourne and Stewart, 1991; Na et al., 2022), the Duke University Religion Index (Koenig and Büssing, 2010), the UCLA Loneliness Scale (Russell, 1996), the Posttraumatic Growth Inventory (Tedeschi and Calhoun, 1996), and the Duke-UNC Social Support Questionnaire (Broadhead et al., 1988). Each captures elements of affective stability, optimism, social connection or meaning-making that contribute to overall resilience. Together, these measures emphasize the multidimensional nature of resilience. Like the other questionnaires discussed, they can only measure resilience if administered repeatedly.

Beyond measurement, resilience research seeks to identify Han and Wangpredictors and trajectories of resilience over time using various approaches including those of Bonanno et al. (2011) and Haag et al. (2022). Longitudinal and lifespan studies in both children and older adults identify protective factors such as emotional regulation, social engagement, and purpose, and demonstrate that resilience evolves across developmental stages (Gooding et al., 2012; Chen et al., 2023; Masten, 2016). Quantitative models like the Challenge Model of Resilience (Suh et al., 2025) found that a moderate level of exposure to Adverse Childhood Events can be protective and build resilience compared to having no exposure, highlighting resilience as a non-linear process.

From an operational and applied perspective, resilience research on military and security personnel has examined outcomes based on third-party and peer observation, and success or failure in training and screening courses (Farina et al., 2019; Mantua et al., 2022; den Hartigh et al., 2025). Selection for elite units such as special operations forces creates standardized high-stress environments that permit concurrent assessment of psychological, biological, and performance outcomes (Beal, 2010; Farina et al., 2019; Farina et al., 2025). Similarly, clinical diagnostic tools can measure resilience by assessing outcomes such as depression, anxiety disorders, and posttraumatic stress disorder (PTSD), after a stress exposure (Kalin, 2021; Srivastava et al., 2024), with the absence of disorder indicative of resilience mechanisms at work. Jones et al. (2022) provide a comprehensive review of the literature on the use of mental health outcomes.

### 2.6. Challenges and opportunities for advancing validated psychosocial resilience metrics

Although there is no widely held consensus on how to optimally measure psychosocial resilience, several tools, such as the CD-RISC, the CYRM, the Grit scale, and the Brief Resilience Scale (Smith et al., 2008), are well-validated and have similar reliability. Other measures of mental health, such as semi-structured interviews that detect various mental conditions. Typically, the Structured Clinical Interview for DSM Disorders is used as it provides detailed information on mental status post-stress and is well-validated. Research conducted by military and security communities uses simulations of high-stress environments, such as survival courses that include simulated prisoner of war-like environments, which are often designed to distinguish individuals who respond successfully and are resilient and others who fail (Beal et al., 2010; Hardy et al., (2010); Farina et al., (2019); Farina et al., (2025); (Mantua et al., 2022; den Hartigh et al., 2025). These types of studies provide an opportunity to validate questionnaires such as the CD-RISC and typically include other direct and indirect measures of resilience, such as demographic, psychological, biological, and genetic measures.

Despite considerable progress, several barriers constrain the standardization and application of psychological resilience metrics. Despite NIH’s development of a unified definition of resilience (Brown et al., 2023a), the existence of many valid domain-specific definitions and the inconsistent application of these definitions within and across domains is a significant barrier in studying and measuring resilience. The recent comprehensive review of Wolke et al. (2025) noted that most psychosocial resilience studies (68%) did not explicitly define resilience. This lack of definitional consensus, or recognition of a unified definition such as the NIH definition, introduces variability in study design, measurement approaches, data interpretation, and the translation of findings into implementation, clinical practice, and public policy.

Another barrier that also presents a research opportunity, is the need to understand how current and emerging metrics and measures of psychosocial resilience apply to digital and online interactions. This is especially critical for younger individuals, whose experiences with digital communities vary widely and may uniquely influence their psychosocial development and resilience trajectories (Singleton et al., 2016; Panter-Brick et al., 2022).

Opportunities to improve the study of psychological resilience exist through the application of new research and analysis methods. Research on psychosocial resilience has used quantitative, qualitative, and mixed methods designs to contextualize resilience trajectories and to study socially marginalized populations using individual accounts of factors associated with coping and thriving under stress. Inclusion of participatory, qualitative methods, including arts-based visual methods, has increased understanding of global populations that report culturally specific coping mechanisms that standard scales may not capture (Haffejee and Theron, 2018). Further development of contextual and population-focused methods and the application of more sophisticated data analysis techniques, including network analysis and artificial intelligence, will be critical for connecting data on individual life course to patterns of multi-system resilience (Cameron et al., 2013; Jefferies et al., 2022). Comprehensive overarching reviews, such as that of Wolke et al. (2025), that conceptualize resilience research and evaluate methods are particularly useful.

Psychosocial resilience is shaped not only by individual attributes and coping processes, but also by the social and structural contexts in which individuals live. Many constructs assessed at the individual level—such as perceived support, adaptability, and recovery following adversity—have direct parallels at the community level, where social cohesion, resource availability, and collective recovery influence resilience outcomes. These shared constructs highlight opportunities for harmonizing measures across individual and community domains.

#### 3.0: Measures and metrics of community resilience

Community resilience reflects the integrated impact of physical and behavioral health, economic stability, community services and physical infrastructure, and social networks and engagement, on individuals and community systems. Physical and behavioral health and access to services enhance the ability of the population to recover from challenges and, thus, contribute to community resilience. Economic stability, housing, education, and community-wide social values that influence health strengthen community resilience by reducing vulnerability for all residents. Established social connections, networks, and community engagement create an environment of trust, collaboration, and mutual support that is critical when responding to a community challenge that fosters community well-being (Akintobi et al., 2025).

The definition of community can vary widely across different contexts. For the NIH Workshop and this article, community is defined as the way in which people are bound together geographically, culturally, economically, or politically. A community’s identity can be shaped not only by its geographic location but also by shared cultural heritage, social networks, and common economic or political interests. The unique and multidimensional characteristics of each community must be considered when measuring community resilience.

While the measurement of community resilience is useful for research purposes, it is most often used to inform community-level policy and funding decisions that strengthen communities (The National Academies Press, 2012; National Risk Index, 2021). The multidimensional nature of communities necessitates the intentional selection and use of combinations of measures and metrics to create models, indices, or frameworks that allow community leaders to measure community health or function before and after a challenging event. These tools also help identify factors that contribute to a community’s ability to resist an event, and resources available to support recovery and growth (Links et al., 2018; Ellis et al., 2022, Zuzak, 2023).

**Fig. 2.**
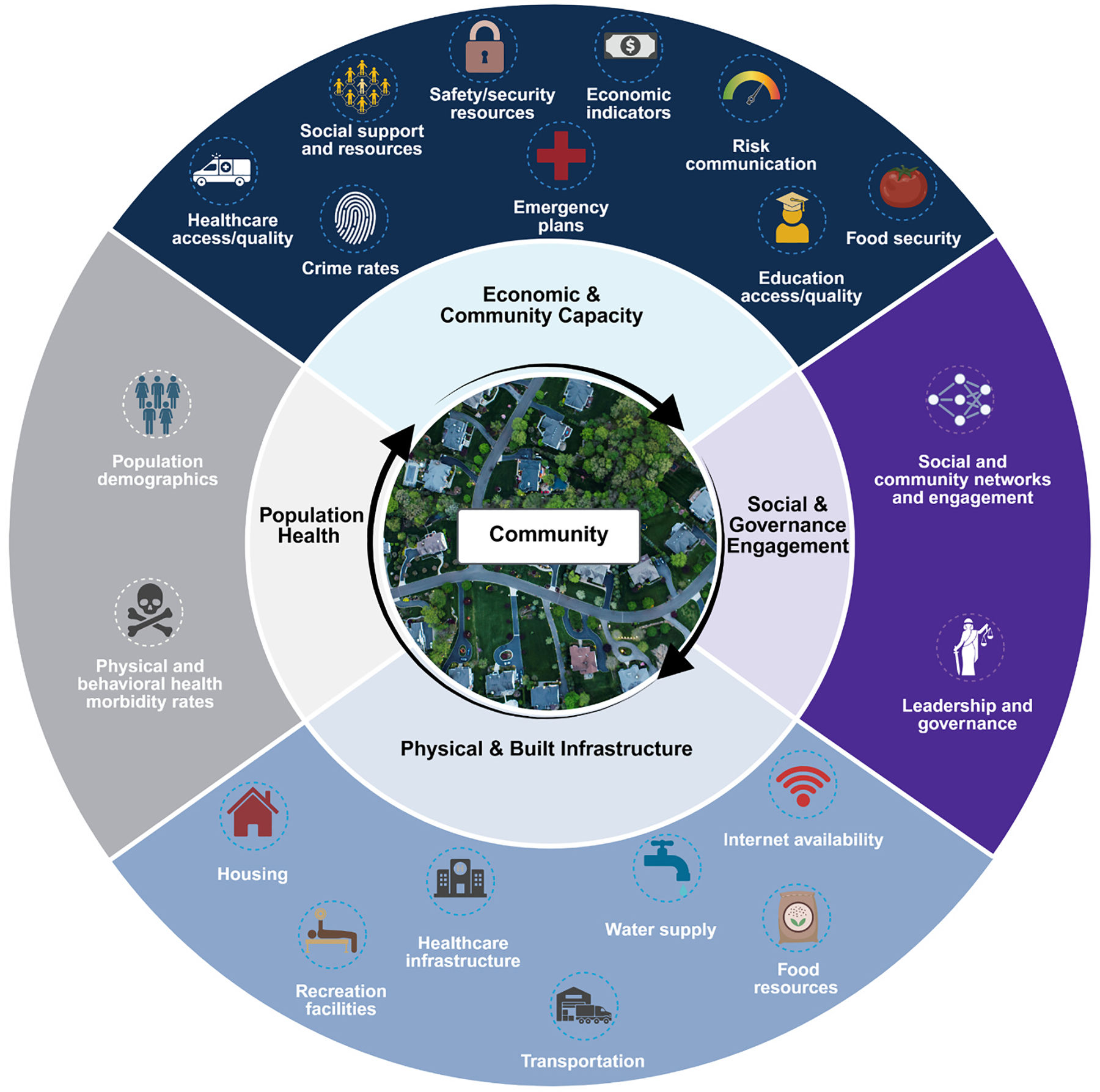
Community resilience metrics by subdomains. Commonly used categories of community resilience metrics are organized by subdomains of population health, economic and community capacity, physical and built infrastructure, and social and governance engagement.

Although adverse events affecting community resilience have historically focused on environmental disasters, other community stressors include health crises (e.g., COVID–19 pandemic), economic downturns, high crime rates, and industry changes. These events may occur at a single time (e.g., a mass shooting) or may be ongoing, such as the long-term impact of inflation or high unemployment rates on a community. Measuring community resilience first requires defining its characteristics and establishing a baseline. The metric categories presented in Fig. 2 can be used to describe a community at baseline, during, or after an adverse event. They may be used individually to highlight specific characteristics or in combination to provide a comprehensive view of the community and its capacity to respond to adversity.

More than 90 community resilience measurement tools exist, most originally designed for disaster or environmental resilience (Zuzak et al., 2023). Even among these tools, there is considerable disparity in the type of disaster or stressor addressed, the metric domains included, and the specific indicators used to measure a community’s response to an event. A review of 33 commonly used measurement approaches found that few consider all metric domains, highlighting the need for further work to identify the most effective strategies for measuring community resilience for both research and practical applications (National Academies of Sciences, Engineering, and Medicine, 2019).

Several established frameworks group metrics into structural, functional, and integrative categories. Structural metrics emphasize infrastructure and system capacity. For example, the Baseline Resilience Indicators for Communities tool quantifies resilience across six domains—human well-being, economic and financial resources, community capacity, institutions and governance, infrastructure and housing, and environmental and natural resources (Habets and Cutter, 2024). Similarly, the City Resilience Framework defines four dimensions that reflect the systems supporting health, social, and financial stability, critical services, and inclusive decision-making in urban environments (Silva, 2025). The Community Resilience Estimates, developed by the U. S. Census Bureau, track neighborhood-level social vulnerability and the capacity of households to absorb and recover from disasters like COVID–19 (U.S. Census Bureau, 2024). The COPEWELL model offers a systems-level view by integrating data across domains such as communication, education, healthcare, and public health to evaluate both resistance and recovery (Links et al., 2018). Together, these tools operationalize resilience as a multidimensional construct including human, institutional, and environmental subsystems.

Other frameworks capture how community processes affect resilience outcomes. The Community Resilience Framework demonstrates how sector-specific policies, such as those in housing, education, and criminal justice, interact to influence outcomes like employment, homelessness, and health (Ellis et al., 2022). The Conjoint Community Resiliency Assessment Measure uses a 10-item survey to estimate resilience based on leadership, preparedness, social trust, and place attachment (Leykin et al., 2013). The Community Mobilization Measure evaluates shared concerns, leadership, and social cohesion within six domains of mobilization (Lippman et al., 2016). Lastly, the Resilience Capacity Index provides a composite score based on twelve standardized indicators across economic, sociodemographic, and connectivity domains, allowing for comparison across 361 U.S. metropolitan regions (Teaman, 2011). Together, these tools reflect the diversity of approaches available to assess and strengthen community resilience across different contexts and scales.

### 2.7. Challenges and opportunities for advancing validated community resilience metrics

The study of community resilience struggles with a lack of up-to-date statistical data at the community level (National Academies of Sciences, Engineering, and Medicine, 2019), the potential lack of expertise in qualitative data collection and analysis, and the insufficient validation of many existing tools. To some degree, these challenges are not surprising, as the disparate metrics reflect different research domains, are used for various purposes, and validation assessments vary across tools, if used at all (Gu et al., 2023).

Although community resilience tools often use quantitative data to produce an index, few methodological descriptions include indexing guidelines such as testing for sensitivity and internal consistency and data procedures (e.g., weighting, normalization, missing value replacement) (National Academies of Sciences, Engineering, and Medicine, 2019). Additionally, most efforts do not measure change over time and are used to establish point-specific resilience to a specified acute incident; thus, they have no temporal capacity.

While a variety of tools are currently employed to assess community resilience, the lack of standardized and validated measures and metrics remains a significant challenge for research, particularly when designing studies that aim to capture the multifaceted nature of community resilience within constrained funding environments. As Cutter (2016) emphasizes, communities vary widely in their physical, social, and built-in environments; governance structures; social infrastructure; capacities; and the stressors they encounter. For example, the measures and metrics needed to assess the resilience of a coastal community vulnerable to hurricanes differ from those needed to assess the resilience of an inland rural community grappling with economic issues. Rigid frameworks risk overlooking these differences, while flexible, mixed-method designs provide a more accurate approach that combines quantitative and qualitative methods. Quantitative sources—census data, public health surveillance, or infrastructure inventories—can be complemented by qualitative data from focus groups, interviews, participatory observation, community member surveys, case studies, and reviews of written documents (U.S. Department of Health and Human Services, 2011). For instance, while statistical data might indicate sufficient hospital capacity, interview data may reveal that residents feel unsafe using them due to perceived discrimination. Thus, effective resilience measurement requires both multisectoral indicators and community participation in defining and interpreting them.

Community participation and trust in the data collection process directly affect the reliability of findings, as low engagement can result in incomplete or biased information. Tools such as the Community Engagement Measure (Goodman et al., 2017) can be used to quantify and assess community involvement in research, underscoring the need for participatory approaches in community resilience science. Building community ownership in data collection enhances both the accuracy and legitimacy of findings and serves to strengthen community resilience through the process.

Many communities are already using various community-level resilience metrics to improve the health and well-being of their members, with measurement evolving from static, disaster-focused metrics to system-based frameworks that reflect health, equity, and adaptability. While the variety of metrics reflects the complexity of community systems, resilience science will be strengthened by the development of clearer definitions of community and the harmonization of metrics for comparative evaluation across populations and events. Standardizing analytic procedures while using flexible mixed method approaches that reflect heterogeneous communities will lead to the development of realistic strategies that promote community resilience.

Community resilience is closely intertwined with environmental systems that shape exposure, vulnerability, and recovery capacity. Measures of community function, infrastructure, and social organization often intersect with environmental indicators such as built environment integrity, access to green space, and system redundancy. Considering these domains together allows for examination of how they jointly support or constrain resilience of the community as a whole and of the individuals within it in the face of acute and chronic challenges.

### 2.8. Measures of environmental resilience

The intersection of environmental systems and population health has become critically important as climate-related disasters become more frequent and severe. Events such as Hurricane Katrina’s devastation of New Orleans in 2005 and the central Texas floods in 2025 that exposed inadequate warning systems and under-resourced infrastructure demonstrate how environmental resilience directly shapes public health outcomes. Environmental resilience, originally conceptualized in engineering and environmental sciences, refers to a system’s ability to absorb shocks while maintaining core functions and structural integrity (Nelson et al., 2007). It is characterized by structural stability under stress, self-organization during disruption, and adaptive learning that enables improved responses to future challenges. These features are essential not only for ecosystems or built infrastructure, but also for the environments in which people live, work, access healthcare, and build communities.

The application of environmental resilience concepts in public health research remains significantly underdeveloped (Mosadeghrad et al., 2023). This gap becomes particularly problematic when climate-related disasters increasingly test both infrastructure and institutional capacities, exposing long-standing inequities in housing, infrastructure, and social vulnerability. Nelson et al. (2007) emphasize that resilience must be understood as an emergent property of linked social-ecological systems rather than isolated structures, requiring coordinated adaptation across multiple levels and temporal scales.

Efforts to quantify environmental resilience have advanced from conceptual frameworks to specific, testable measures, models, and tools. Structural integrity, infrastructure redundancy, green infrastructure, and built environment health indicators constitute the primary categories. Structural integrity metrics assess the robustness of built infrastructure through load-bearing analyses, seismic ratings, or flood protection standards. Hospitals designed to withstand a magnitude 7.0 earthquake exemplify infrastructure-level resilience that preserves healthcare continuity. These metrics lend quantitative rigor but often emphasize physical infrastructure at the expense of social dimensions of resilience. Redundancy metrics assess whether backup systems exist that can maintain health-supporting functions when primary systems fail. This includes emergency power for medical facilities, protected and redundant IT resources required for patient care, alternative transportation routes, and distributed versus centralized service delivery. Greater redundancy equates to greater resilience capacity due to the presence of multiple pathways for maintaining essential health services.

Natural systems also serve as green resilience infrastructure. Measures of stormwater management capacity, air pollution reduction, and urban heat island mitigation quantify ecological buffering functions. Research demonstrates that green infrastructure provides multiple ecosystem services, including flood risk reduction, water quality improvement, and climate resilience benefits (Dhakal and Chevalier 2016; Natural Resources Defense Council, 2024). Programs like Philadelphia’s *Green City, Clean Waters* evaluate “gallons of stormwater managed per dollar invested,” directly linking ecological interventions to resilience outcomes (U.S. Environmental Protection Agency, 2024). Communities with tree canopy coverage exceeding 40 % show significantly greater thermal resilience during extreme heat events, and systematic reviews indicate that green infrastructure provides other benefits such as increased property-value (Clapp, 2019).

Mobility and accessibility contribute another dimension. Algorithmic tools such as the Walk, or Bike scores assess mobility resilience, i.e., the capacity for community members to access essential resources despite transportation disruptions. The Walk Score ranges from 0 to 100 and integrates distance to amenities, intersection density, and block length. Higher scores correlate with reduced obesity, increased physical activity, and annual healthcare savings (Wang et al., 2023; Creatore et al., 2019). These scores standardize comparisons across regions but may not capture pedestrian safety, accessibility for individuals with disabilities, or actual walking behavior. Additionally, their applicability is limited in rural areas, where infrastructure and land use patterns greatly differ.

Systems-based models expand measurement beyond static indicators. The risk-exposure-mitigation (REM) model links hazard characteristics, social vulnerability, and adaptive capacity, framing resilience as the interaction among risk, exposure, and mitigation. Acute hazards such as floods, wildfires, and heatwaves, and chronic stressors such as pollution, unsafe housing, and food insecurity are evaluated simultaneously. An example of a REM model is the RAIDAR-ICE model that assesses chemical exposure and risk (Li et al., 2018). Similarly, Bruneau et al.’s (2003) R4 framework of robustness, redundancy, resourcefulness, and rapidity quantifies a system’s recovery capacity using measures such as Mean Time to Recovery (MTTR) and recovery rate validated for critical infrastructure by the National Institute of Standards and Technology (National Institute of Standards and Technology, 2017). For healthcare systems, shorter MTTR values, such as rapid restoration of hospital function after a disaster, signal greater resilience.

FEMA’s HAZUS methodology specifically measures adaptive capacity dimensions of environmental resilience by quantifying displacement, debris, and shelter requirements and has been extensively validated through post-disaster damage surveys and insurance loss data across multiple hazard types (Scawthorn et al., 2006; Vickery et al., 2006). For example, HAZUS can estimate that a 7.0 earthquake in Los Angeles would displace 270,000 households and require 1400 emergency shelters, directly measuring the community’s absorption capacity and recovery resource requirements. Recent applications have demonstrated HAZUS’s effectiveness in predicting flood losses and informing climate resilience planning (Federal Emergency Management Agency, 2024). However, despite its utility, HAZUS operates at a relatively coarse spatial resolution and often lacks meaningful community input in parameter setting. Complementary agent-based models, such as Infra-SIM simulate cascading failures across interconnected systems. For example, models can test how a 40 % power grid failure affects hospitals and transportation networks. These models illuminate interdependences and redundancy but require access to extensive, high-resolution local data and significant computational resources rarely available to smaller communities.

Composite metrics further integrate environmental, digital, and infrastructural factors. The Centers for Disease Control and Prevention’s (CDC) Community Resilience Estimates combine broadband access, green-space coverage, and built environment factors such as housing stability into multidimensional resilience scores (Centers for Disease Control and Prevention, 2024). Digital connectivity is recognized as essential for emergency communication, while environmental metrics capture ecological buffering capacity. This multi-sectoral approach reflects the World Health Organization’s (WHO) emphasis on climate-resilient health systems (Mosadeghrad et al., 2023; World Health Organization, 2023). Its strengths include standardized methods and national coverage, but equal weighting of domains may not reflect local priorities, and its county-level resolution may obscure critical neighborhood-level variation.

Similar to community resilience, tools have been developed to help communities assess environmental resilience or to plan for the future. The Climate Resilience Measurement for Communities tool uses a systems-based approach integrating physical, financial, human, social, and natural systems (Rubenstein et al., 2024). Originally designed by both practitioners and researchers as part of the Zurich Flood Resilience Alliance, it was developed to measure community-level floods or heatwaves but can include diverse hazards by tracking measures such as school attendance, permeable surface area, energy system robustness, and financial support access. Continuous environmental monitoring enhances adaptive management. The U.S. Environmental Protection Agency’s (EPA) Air Quality Index (AQI) and urban heat maps provide baseline measures of exposure. AQI values exceeding 100 or heat differentials above 5 °F signal diminished resilience capacity and trigger public health interventions. These systems are costly and may fail to capture neighborhood-level variations. Municipal dashboards, such as the New Orleans Municipal Building Energy Use Dashboard (City of New Orleans, 2025), track pump station status, emergency shelter capacity, and backup power availability, operationally enhancing situational awareness but limiting cross-city comparability and requiring sustained investment.

Participatory and data-driven methods increasingly complement technical tools. Geographic information systems (GIS) and tools like mySidewalk engage residents in mapping environmental assets and vulnerabilities (Luo and Wang, 2003; Elwood, 2006). Crowdsourced digital reports on broken streetlights, flood-prone areas, or air quality help communities create a local map of community-identified resilience assets and vulnerabilities but depend on continuous participation and data quality control and may reflect bias toward more engaged residents.

Social media sentiment mapping offers a low-cost means of monitoring perceived environmental safety. Natural language processing of algorithms analyzes geotagged posts for environmental concerns, and studies demonstrate moderate correlations between negative environmental sentiment and emergency department visits, suggesting that positive community perceptions reflect higher psychosocial environmental resilience (Gaskin et al., 2021).

### 2.9. Challenges and opportunities for advancing validated environmental resilience metrics

Despite growing sophistication, most environmental resilience metrics remain poorly validated against health outcomes (Clapp et al., 2019). Few studies directly link environmental resilience indicators—such as green infrastructure or system redundancy—to measurable improvements in morbidity or mortality, limiting predictive utility and policy adoption. Moreover, environmental resilience operates across multiple time scales, requiring metrics that capture both acute/rapid response and chronic/long-term adaptive capacity. Technical resilience metrics often overlook distributional equity: rapid recovery in affluent neighborhoods may coincide with prolonged disruption in disadvantaged areas. Addressing this requires embedding equity analytics into resilience frameworks to evaluate who benefits from resilience investments and who remains at risk (Mosadeghrad et al., 2023).

Another challenge lies in balancing comprehensive assessment with feasibility. Sophisticated frameworks require extensive data collection, modeling capacity, and financial resources, which can overwhelm under-resourced jurisdictions, necessitating scalable approaches that maintain rigor without overwhelming local capacity. Additionally, environmental and health interdependencies vary across geographic, cultural, and socioeconomic contexts, requiring adaptation of measurement approaches to local conditions. There is a need for validated composite metrics validated across diverse populations, along with validated composite indices that integrate multiple resilience dimensions and permit longitudinal benchmarking across communities.

Finally, advancing environmental resilience science requires robust intervention development and testing. This includes designing interventions that address multiple resilience domains simultaneously, testing their scalability across diverse settings, and assessing both health and equity impacts of environmental resilience interventions.

### 2.10. Toward a unified framework for measuring resilience across domains

The NIH Resilience Workshop proceedings highlight the need to develop measures and metrics that not only capture resilience within specific domains but also enable comparison and integration across them. As outlined in this article, resilience research spans cellular, physiological, psychosocial, community, and environmental—each defined by distinct methodological and measurement traditions. This diversity reflects the intrinsic complexity of resilience as a scientific construct, which complicates synthesis, comparability, and translation.

Across these domains, recurring constructs such as resistance to perturbation, time and completeness of recovery, and long-term adaptation or growth consistently surface. These elements align closely with the NIH definition of resilience and provide a foundation for identifying measures applicable across diverse contexts, even when operationalized at different scales or through distinct methodologies. Table 4 illustrates examples of cross-domain metrics that demonstrate conceptual convergence. For instance, Time to Recovery (TTR) applies to processes as varied as cellular repair, physiological stabilization, psychosocial adaptation, and infrastructure restoration. Similarly, mobility and access measures—such as the 6-minute walk test, Walk Score, and Healthcare Access Index—reflect both individual capabilities and the broader systems that sustain them.

Despite these shared constructs, many existing measures assess static states or risk profiles rather than dynamic responses to challenge. As summarized in Table 5, research gaps extend across conceptual, methodological, and translational dimensions. These include limited use of challenge-based and longitudinal designs, insufficient validation of resilience-specific indicators, and a lack of harmonized analytic frameworks that support cross-study and cross-population comparison.

Closing these gaps will require continued methodological refinement and consistent application of the NIH resilience framework—including resistance, recovery, growth, and adaptation—to enhance conceptual clarity and support alignment across study designs. Progress will depend on increasing the use of longitudinal and challenge-based approaches that define response trajectories over time and on improving methods for quantifying meaningful change in resilience-relevant outcomes.

Strengthening the validation of measures explicitly as indicators of resilience is another essential step. Priorities include standardizing measurement protocols, identifying meaningful thresholds or minimal clinically important differences, and harmonizing analytic strategies. Integrating cellular, physiological, and psychosocial measures within unified study designs can illuminate mechanistic pathways that contribute to resilience and strengthen the interpretation of functional outcomes. Multiscale approaches that connect acute responses with long-term adaptations will further enhance understanding of resilience across temporal levels.

Extending resilience research beyond individual domains remains critical for developing a unified framework. As discussed in the community and environmental sections, mixed-methods research, longitudinal measurement, and participatory approaches are vital for capturing dynamic change, contextual influences, and population-level variability in recovery and adaptation. Consideration of equity and differential vulnerability is also essential, especially when evaluating community and environmental resilience, where aggregate metrics may obscure disparities in exposure, access, and recovery.

Taken together, the cross-domain measures outlined in Table 4 and the research gaps and needs identified in Table 5 illustrate both the progress achieved and the challenges that remain in building a cohesive science of resilience. While domain-specific advances continue to mature, further alignment of concepts, measures, and study designs across individual, community, and environmental levels will be necessary to strengthen interpretability, comparability, and translational value.

## 3. Conclusion

This article proposes a conceptual roadmap to guide future research. Key directions include mapping domain-specific metrics to shared resilience constructs, identifying methodological overlaps, addressing validation gaps, and developing composite indicators that integrate individual, community, and environmental dimensions. Together, these efforts provide the basis for a coordinated, cross-domain research agenda and for building the methodological, computational, and collaborative infrastructure required to operationalize a validated, unified resilience measurement framework.

Sustained progress will depend on coordinated investment in measurement infrastructure, validation studies, and collaborative mechanisms that bridge domains. Leadership and support from public agencies, private foundations, academic institutions, and industry partners will be vital. Through these efforts, resilience science can advance beyond domain-specific silos to inform interventions, policies, and systems designed to enhance resilience within a shared and measurable framework.

## Figures and Tables

**Table 1:** Chronic mechanistic metrics and measures.

Biological Level	Resilience Process	Representative Metric	Common Measurement Approaches
Cellular	Genomic stability	Genomic instability / homologous recombination deficiency score	Copy number variation; DNA structural changes; damage signatures
	Telomere length	Peripheral blood mononuclear cell telomere length	Flow cytometry, qPCR-based or Southern blot-based assays
	Epigenetics	Differential DNA methylation patterns	Differentially methylated regions/sites profiling
	Proteostasis	Protein quality control	Chaperone activity; proteasome function assays
	Autophagy	Autophagic flux	Autophagy flux assays (e.g., LC3-II–based readouts)
	Nutrient sensing	Expression/phosphorylation/activity	Carbohydrate: insulin/IGF-I, AMPK, SIRT, GCK Protein: mTOR, insulin/IGF-I Fatty acids: GPR40/120, CD36 Cholesterol: SCAP
	Mitochondrial function	Oxidative phosphorylation capacity	High-resolution mitochondrial respirometry
	Cellular senescence	Senescence burden	SA-β-gal activity; cell-cycle arrest markers; SASP profiling
	Stem cell capacity	Regenerative signaling capacity	DNA damage response; growth factor signaling; apoptosis signaling; scRNA-Seq; ATAC-Seq; ChIP-Seq
Tissue	Extracellular matrix remodeling	Structural ECM alterations	Collagen/elastin imaging; matrix metalloproteinase activity
	Intercellular communication	Cell-to-cell signaling	Ligand–receptor analysis; gap junction assays; extracellular vesicle profiling; exosome transfer assays
Systemic	Inflammation	Elevated inflammatory burden	Multiplex cytokine profiling
	Microbiome balance	Altered microbial community structure	PCR-based dysbiosis indices; GA-map assays

**Table 2:** Acute mechanistic stress response and representative resilience metrics and measures.

Stressor	Biological Response	Representative Metric	Common Measurement Approaches
Oxidative stress or hypoxia	Redox and proteostatic stress	Oxidative stress burden	ER unfolded protein response (UPR) stress assay; glutathione assay; hydrogen peroxide assay
Thermal stress	Proteotoxic and unfolded protein response	Heat shock response magnitude	Heat shock protein expression; integrated stress response assays; ER UPR stress assay; cell morphology
Inflammatory challenge	Stress-induced inflammatory signaling	Acute cytokine activation	Cytokine signaling / transcription factor expression / downstream gene expression; ER UPR stress assay; integrated stress response assay
Chemical or toxin exposure	DNA and ER stress activation	Cellular stress response index	Oxidative stress assays; DNA damage assay; ER UPR stress assay; toxicogenomic profiling

**Table 3:** Physiological resilience metrics are categorized by temporal scale and subdomains.

Temporal Scale	Physiological Subdomain	Metric	Measures Reflect	Validation Status
Acute	Cardiopulmonary	Heart rate variability	Autonomic nervous system response to stress	Validated for clinical and research settings
	Cardiopulmonary	Heart rate recovery	Time taken for heart rate to return to baseline post-exercise	Widely used in exercise physiology
	Endocrine	Cortisol	Immediate stress response	Standard biomarker for acute stress
	Neuromuscular	Grip strength	Immediate muscular strength	Simple, validated measure
	Cardiopulmonary	VO2max	Maximum oxygen uptake	Gold standard for aerobic fitness
Chronic	Cardiopulmonary	Blood pressure regulation	Stable blood pressure regulation	Standard clinical measure
	Cardiopulmonary	Maximum inspiratory pressure	Strength of the inspiratory muscles, particularly the diaphragm	Emerging as resilience biomarker
	Metabolic	HbA1C levels	Reflects average blood glucose over 3 months	Standard diabetes diagnostic and management tool
	Metabolic	Lipid profiles	Includes HDL, LDL, and triglycerides	Routine clinical assessment
	Neuromuscular	6-minute walk test	Measures functional exercise capacity	Validated in various populations
	Sleep	Actigraphy, self-report Questionnaires, and polysomnography	Monitors sleep patterns and disturbances.	Widely used in sleep research.
	Aging	Telomere length	Indicator of cellular aging	Emerging biomarker; requires further validation
Long-Term	Aging	Epigenetic clocks (e.g., Horvath Clock)	Estimates biological age based on DNA methylation	Promising tool; ongoing research
	Function	Frailty index	Composite measure of vulnerability in older adults	Validated in geriatric populations

**Table 4:** Sample cross-domain measures and metrics of resilience aligned with defined system responses to stressor exposure.

Measure / Metric	Mechanistic	Physiological	Psychological	Community	Environmental
Grip Strength	–	Musculoskeletal strength, frailty	Persistence, self-efficacy	Frailty indices in aging populations	–
Heart Rate Variability (HRV)	–	Autonomic stress response, recovery	Emotion regulation, PTSD outcomes	Cohort-level stress resilience	Analogous to exposure response (heat, AQI stress)
Sleep Metrics (actigraphy, polysomnography, surveys)	–	Circadian rhythm, metabolic recovery	Mood, cognitive function, stress buffering	Community factors impacting sleep	Impacted by environmental noise, heat, pollution
Mobility/Access Measures (Walk Score, Healthcare Access Index)	–	Gait speed, mobility resilience	Confidence in independence	Social inclusion, transport access	Walkability, redundancy of transport/healthcare
Frailty Index	Senescence markers, proteostasis decline	Multisystem decline (mobility, health)	Includes cognitive/mood items	Population frailty prevalence	–
Oxidative Stress Measures	Glutathione, ER stress assays	ROS balance, antioxidant capacity	Psychological stress links	–	Air pollution/chemical exposures
6-Minute Walk Test (6MWT)	Mitochondrial activity, myocyte health, capillary density and quality	Functional exercise capacity, cardiopulmonary reserve	Motivation and self-efficacy	Community members’ mobility	Ability to walk, exercise, and adhere to physical activity behavior in one’s environment
Cortisol / Stress Biomarkers	UPR assays, cytokine signaling	Acute HPA-axis response	Dysregulation in depression, trauma	Disaster/recovery population stress load	Exposure stress (pollution, climate shocks)
Time-to-Recovery (TTR / MTTR)	Oxidative stress clearance, autophagy recovery	HR recovery, VO_2_ recovery	Psychological recovery time (stress scales)	Health service recovery rates	MTTR for infrastructure, utilities, healthcare systems
Inflammation Markers (cytokines, CRP)	Multiplex cytokine arrays	Systemic inflammation	Stress-related inflammation	Population inflammatory burden	Pollution/heat exposure elevates inflammation

**Table 5:** Priority research gaps for advancing the biomedical science of resilience.

Priority Area	Gap	Why It Matters	Research Need
Foundational Concepts	Inconsistent application of NIH resilience definition	Limits cross-study comparability and synthesis	Standardized use of resist-recover-grow/adapt constructs
Measurement & Metrics	Overreliance on static or cross-sectional measures	Resilience is dynamic and cannot be inferred from single time points	Repeated-measures and challenge-recovery designs
	Limited validation of metrics as resilience outcomes	Many measures reflect health status, not adaptive capacity	Validation against response trajectories following stress
	Lack of Minimal Clinically Important Differences (MCIDs)	Changes may not be meaningful or actionable	Establish MCIDs for key resilience metrics
Mechanistic Resilience	Underrepresentation of mechanisms in human studies	Limits mechanistic understanding of resilience	Integration of cellular assays with human challenge studies
	Poor linkage between acute and chronic mechanistic resilience	Aging and stress responses studied in isolation	Multiscale studies linking acute stress response to biological aging
Physiological Resilience	Lack of standardized protocols	Limits reproducibility and meta-analysis	Harmonized measurement and analytic standards
	Insufficient integration with cellular and molecular data	Physiological metrics lack mechanistic grounding	Multi-omics and physiological coupling studies
Psychosocial Resilience	Proliferation of domain-specific definitions and scales	Reduces coherence across studies	Harmonization around shared resilience constructs
	Heavy reliance on self-report instruments	Limits objectivity and sensitivity to change	Integration of behavioral, biological, and performance metrics
	Limited understanding of digital and online contexts	Major gap for youth and modern stress exposures	Validation of resilience metrics in digital environments
Community Resilience	Many tools lack validation and temporal sensitivity	Cannot assess recovery or growth over time	Longitudinal, mixed-methods community studies
	Insufficient community participation	Reduces relevance, trust, and equity	Participatory and co-designed measurement approaches
Environmental Resilience	Weak linkage between environmental metrics and health outcomes	Limits public health and policy impact	Direct testing of environmental resilience-health pathways
	Limited equity-focused analytics	Masks differential recovery across populations	Equity-explicit resilience metrics
Cross-Domain Integration	Few validated metrics span multiple domains	Prevents unified resilience science	Identification and validation of shared metrics (e.g., time to recovery)
	Limited intervention testing across systems	Interventions remain siloed	Multilevel interventions evaluated across domains
Translation & Implementation	Measurement outpaces implementation science	Slows real-world impact	Scalable, actionable resilience measurement frameworks
